# Carotenoids from UV-resistant Antarctic *Microbacterium* sp. LEMMJ01

**DOI:** 10.1038/s41598-019-45840-6

**Published:** 2019-07-02

**Authors:** Maria Cristina P. P. Reis-Mansur, Janine S. Cardoso-Rurr, Josemar V. Maiworm Abreu Silva, Gabriela Rodrigues de Souza, Verônica da Silva Cardoso, Felipe Raposo Passos Mansoldo, Yuri Pinheiro, Júnia Schultz, Luciene B. Lopez Balottin, Antonio Jorge Ribeiro da Silva, Claudia Lage, Elisabete Pereira dos Santos, Alexandre Soares Rosado, Alane Beatriz Vermelho

**Affiliations:** 1 BIOINOVAR - Biocatalysis, Bioproducts and Bioenergy, Paulo de Góes Institute of Microbiology, Federal University of Rio de Janeiro (UFRJ), Rio de Janeiro, Brazil; 2 LaRBio – Radiations and Biology Laboratory, Carlos Chagas Filho Institute of Biophysics, Federal University of Rio de Janeiro (UFRJ), Rio de Janeiro, Brazil; 3 Labio/Dimav/Inmetro – Laboratory of Tissue Bioengineering/Directorate of Metrology Applied to Life Sciences/National Institute of Metrology, Quality and Technology, Duque de Caxias, Brazil; 4 Research Institute for Natural Products, Federal University of Rio de Janeiro (UFRJ), Rio de Janeiro, Brazil; 5 LEMM - Laboratory of Microbial Molecular Ecology, Paulo de Góes Institute of Microbiology, Federal University of Rio de Janeiro (UFRJ), Rio de Janeiro, Brazil; 6 Faculty of Pharmacy, Galenico Development Laboratory, Federal University of Rio de Janeiro (UFRJ), Rio de Janeiro, Brazil

**Keywords:** Biotechnology, Microbiology

## Abstract

The *Microbacterium* sp. LEMMJ01 isolated from Antarctic soil does not belong to any of the nearest species identified in the RDP database. Under UV radiation (A, B and C wavebands) the survival fractions of *Microbacterium* sp. cells were much higher compared with wild-type *E*. *coli* K12A15. Especially remarkable for an Antarctic bacterium, an expressive resistance against high UV-B doses was observed. The increased survival of DNA repair-proficient *E*. *coli* grown overnight added of 0.1 mg/ml or 1 mg/ml of the whole pigment extract produced by *Microbacterium* sp. revealed that part of the resistance of *Microbacterium* sp. against UV-B radiation seems to be connected with photoprotection by its pigments. Scanning electron microscopy revealed that UV-A and UV-B ensued membrane alterations only in *E*. *coli*. The APCI-MS fingerprints revealed the diagnostic ions for neurosporene (m/z 580, 566, 522, 538, and 524) synergism for the first time in this bacterium by HPLC-MS/MS analysis. Carotenoids also were devoid of phototoxicity and cytotoxicity effects in mouse cells and in human keratinocytes and fibroblasts.

## Introduction

Carotenoids are natural pigments that exhibit many biological functions including antioxidant effects. New sources of antioxidant substances and solar protectors have been the target of worldwide research in cosmetology. The Antarctic Continent is an extreme environment and has a very different microbial biodiversity due to its hostile ecosystems. Numerous pigments produced by microorganisms resistant to ultraviolet radiation (UV) have been found on this continent. Bacteria isolated from Antarctica possess mechanisms to protect themselves from the harsh environmental conditions. Possibly they harbor increased synthesis of photoprotective pigments such as carotenoids, due to the high levels of local radiation. Also, the photoprotective pigments help stabilize membrane structure during growth under low temperatures^1^.

Solar UV radiation causes distinct types of damage: UV-A radiation (400-315 nm) leads to indirect damage to cellular DNA, proteins, and lipids by generating intracellular chemical intermediates such as reactive oxygen species (ROS). This type of UV radiation causes mutagenesis in eukaryotic cells, photoageing and other skin disorders^2^. In contrast, UV-B (315–280 nm) radiation causes direct DNA damage by inducing the generation of DNA photoproducts, such as cyclobutane pyrimidine dimers and the pyrimidine-pyrimidone photoadducts. These rays have been termed as “burning” or “erythemal” rays since they are primarily responsible for the redness associated with sunburn, erythema and skin cancer^3^. The most energetic rays are the UV-C (280–100 nm) wavelengths, under which the main absorption of nucleic acids peaks; however, the ozone layer efficiently filters these UV-C rays, allowing only the passage of UV-B and UV-A^4^. The UV-B pro-oxidant effects are especially critical for environmental bacteria, as demonstrated for the cyanobacteria *Scytonema javanicum*, which, in turn, responds by switching on many antioxidant substances^5^. Agogué *et al*.^6^ suggested that carotenoids from bacteria isolated from Antarctic sea surface microlayers, and their polysaccharides, could be agents determining the resistance of Antarctic bacteria acting as sunscreen substances against solar radiation.

Carotenoids are found in fruits, vegetables, roots, plants and microorganisms including microalgae, other eukaryotes (yeasts and fungi) and genera of the Archaea and Bacteria domains. Extremophilic microorganisms are sources of bioproducts including carotenoids with special properties. In this sense, carotenoids have been studied in halophilic Archaea^7^, in multi extremophile *Deinococcus-Thermus* group^8^ and several other bacteria including the *Bacillus* genus^9^ and *Escherichia coli*^10^.

Carotenoids are framed of an important group of isoprenoid chain, characterized by the presence of a conjugated tetraterpene (C_40_) whose colors range from colorless to yellow, orange, and red. In this family there are over 1100 substances^11^. The conjugated double-bond chain of carotenoids, besides determining their colors, behaves as light-absorbing chromophores playing important biological roles in protecting cells from the damaging effects of UV radiation, as well as exerting antioxidant effects^12^. Briefly, when present in a cell under irradiation, a carotenoid molecule may undergo electronic energy exchange during energy quenching, driving it into a triplet state. Under this configuration, it can easily react with fundamental, triplet oxygen, thus preventing oxidation reactions. Upon returning to its deactivated, single state level, the molecule is also capable to resonantly interact with energy-activated singlet oxygen, thus depleting the cellular medium off this radical species. Even though these reactions act in photoprotective mechanisms, they eventually destroy the carotenoid^13^. Indeed, these antioxidant effects besides their health benefits, also bring profit to the cosmetic sector^14^. These structurally diverse carotenoids harbor other functions such as precursors of vitamin A in animals and humans besides their multiple applications in biotechnology and biomedicine such as safe food colorants, animal food supplements, nutraceuticals and pharmaceuticals as well as important applications in the cosmetic industry^15^. Due to this wide range of applications, carotenoids clocked up a $766 million market in 2007, increasing to $919 million in 2015, with an annual growth rate (CAGR) of 2.3%. By 2018, the carotenoid market is expected to reach $1.4 billion. Beta-carotene itself comprises the largest share in the biotechnological market. The estimated costs of production of natural astaxanthin is around US$700/kg when using microalgae as the raw material, or US$250/kg when using fungi^16,17^ as the biological source.

The presence or absence of oxygen is the criterion to classify carotenoids as xanthophylls (with oxygen) or carotenes, which lack oxygen. Each carotenoid species may assume its own *trans*- and *cis*- isomeric forms^8^. Pigments of synthetic origin have been abolished from human consumption due to their toxic, carcinogenic and teratogenic side-effects. There is a consumer’s tendency to avoid foods containing synthetic pigments which have led food industries to replace them by natural ones. However, new less-toxic synthetic pigments are being developed leading to innovative perspectives^17^. In this sense, microorganisms are now considered as natural sources of biomolecules with potential biotechnological applications in industry using eco-friendly processes. These microorganisms are easily cultivated in bioreactors under controlled conditions of nutrients, pH, temperature and aeration, and can be genetically modified using synthetic biology to improve production of the pigments^18^.

In the present work, a pigmented heterotrophic bacterium isolated from Antarctic nitrogen-rich ornithogenic soils was exposed to cycles of simulated ambient solar radiation to select resistant strains bacteria. The selected *Microbacterium* sp. isolate LEMMJ01 showed a strong resistance against UV radiation, so that its pigments were extracted, and the carotenoids characterized physico-chemically.

## Results and Discussion

### *Microbacterium* sp. isolate LEMMJ01 identification

All isolates were identified, after Gram staining and color detection in colonies, by molecular identification. The identification features for the Antarctic isolates are summarized in Table 1, as well as the comparative effects of UV-B radiation.

**Table 1:** Results of the sequencing the region encoding for 16S rRNA by compararison with GenBank database, Gram staining, color colonies and survival fraction to UV-B radiation.

Isolate number	Closest specie/acess number Genbank	Number of analyzed bases of 16S rRNA gene/percentage of similarity	Gram Staining	Colonies color	Survival fraction to UV-B 312 nm 8 kJ/m^2^ (log^10^)
1	*Planococcus* sp. FN377718.1	1429 pb/99%	+	yellow	1.48e-03
2	*Psycrobacter* sp. FN377742.1	1366 pb/99%	−	yellow	ND
3	*Bacillus amyloliquefaciens* HQ844500.1	1387 pb/98%	+	yellow	1.58e-01
4	*Arthrobacter psychrochitiniphilus* AB588633.1	1382 pb/99%	−	yellow	ND
5	*Microbacterium* sp. AM423149.1	1921 pb/97%	+	orange	4.39e-01
6 Positive control	*Escherichia coli* K12A15	Wild type	−	white	4.49e-05

ND = non detected survival.

The soil processing protocol was described in a previous work^19^ predicting the biotechnological potential of bacteria isolated from Antarctic soils, including the rhizosphere of vascular plants. Single colonies of the psychrophilic bacteria were isolated on LB medium after growing at 12 °C for 48 h. These isolated colonies were then grown, and their genomic DNA was extracted using the FastDNA SPIN kit for soil (MoBio) and quantified using a Qubit fluorometer (Thermo Fisher Scientific). The Nextera XT DNA library kit was used to build paired-end 250-bp libraries, which were sequenced on an Illumina MiSeq. Assembly was performed by MR DNA (Shallowater, TX) using NGen version 12 from DNAStar, and the coding DNA sequences (CDSs) were predicted and annotated using two tools, the NCBI Prokaryotic Genome Automatic Annotation Pipeline (PGAAP) and the Rapid Annotations using Subsystems Technology (RAST) server^20^.

*Microbacterium* sp. isolate LEMMJ01 was selected due to its remarkable vivid colony color and its higher resistance to UV-B radiation when compared with the standard strain, *E*. *coli* K12A15. *Microbacterium* sp. is an actinomycete that is widespread in nature and can be found in diverse and extreme environments such as Antarctica, desertic soils, and in oil refinery plants^21,22^. A number of species in this genus are known species with several biotechnological applications synthesizing bioproducts including amylases and other enzymes capable of degrading multiple polycyclic aromatic hydrocarbons and biosurfactants^23^.

**Figure 1:**
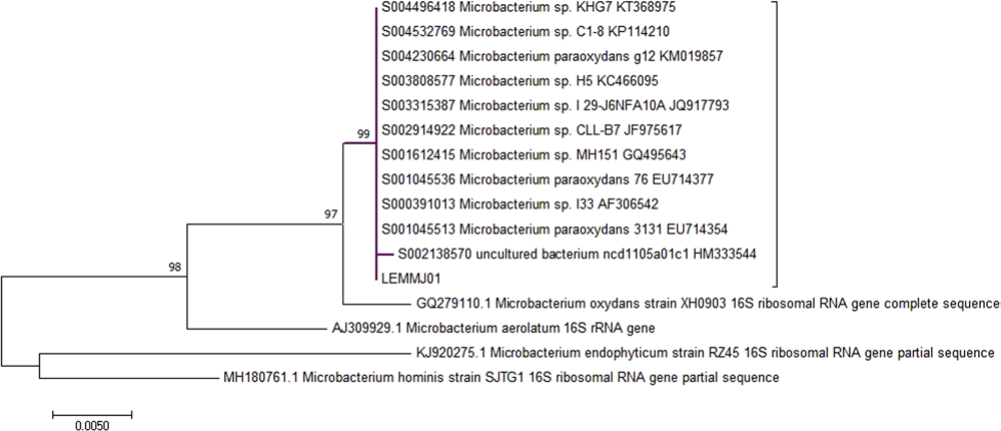
Molecular phylogenetic analysis. The tree with the highest log likelihood (−3555.2025) is shown. The percentage of trees in which the associated taxa clustered together is shown next to the indicated branches. Initial trees for the heuristic search were obtained automatically by applying Neighbor-Joining and BioNJ algorithms to a matrix of pairwise distances estimated using the Maximum Composite Likelihood (MCL) approach, and then selecting the topology with the superior log likelihood value. The tree is drawn to scale, with branch lengths measured in the number of substitutions per site. The analysis involved 12 nucleotide sequences. All positions containing gaps and missing data were eliminated. There were a total of 1266 positions in the final dataset.

Phylogenetic analyses based on 16S comparisons showed that isolate LEMMJ01 does not belong to any of the closest species identified in the RDP database, *Microbacterium paraoxydans* and *Microbacterium oxydans* (Fig. 1). So, it is likely that this isolate is a new carotenoid-producing species, taking into account its position among a number of non-described, unculturable sequences. Recently, our group sequenced the genome outline of isolate LEMMJ01, and a number of key genes involved in C50 carotenoid biosynthesis were found as a cluster that included genes encoding C50 carotenoids C50 cyclases^24^. Genomic data also reinforced the possibility that a new species of *Microbacterium* is being reported in this study. This will be investigated further with a polyphasic taxonomic approach.

Previous work by Trutko *et al*.^25^ detected isoprenoid pigments in 71 out of the 78 strains belonging to the family Microbacteriaceae. In addition, eight out of the sixteen strains of the genus *Microbacterium* are able to synthesize neurosporene, a precursor of lycopene and β-carotene.

In the actinomycete *Microbacterium arborescens*-AGSB the pigment was identified as a lycopene-type carotenoid^26^. The structure of a pigment from *Microbacterium oxydans* was characterized by Meddeb-Mouelhi *et al*.^27^. The pigment was found to have the molecular formula of C_27_ H_42_ O_2_ with the chemical structure (8-hydroxymethyl-2,4,12-trimethyl-14-(2,6,6-trimethyl-cyclohex-2-enyl)-teradeca-3,7,9,11,13-pentan-2-ol). Carotenoid pigments from *Microbacterium paraoxydans* were isolated and characterized by Ojha *et al*.^28^, and the finding of a neurosporene structure was also proposed.

### UV radiation resistance

Antarctica is a continent with extreme environmental conditions for life to thrive and this limitation leads to survival adaptation mechanisms in microorganisms^29^. One of the extreme environmental conditions in the Antarctic environment is the high incidence of UV which is a natural selective factor, and which favors the appearance of microorganisms resistant to solar radiation. In the 1980s, the ozone hole in Antarctic region was described and was reported to cause a high amount of ultraviolet (UV) rays over the region. This effect is intensified by the ice-generated reflection^30^. UV-B radiation is strongly attenuated by the ozone layer and the ozone hole produced a harmful impact on Antarctic life. In the spring, over 50% of the ozone column can be depleted, leading to a doubling of UV-B levels^31^. When present in above normal levels, UV-B can damage the DNA of all organisms and inhibit photosynthesis in marine microorganisms reducing productivity by 5–20%^32,33^.

**Figure 2:**
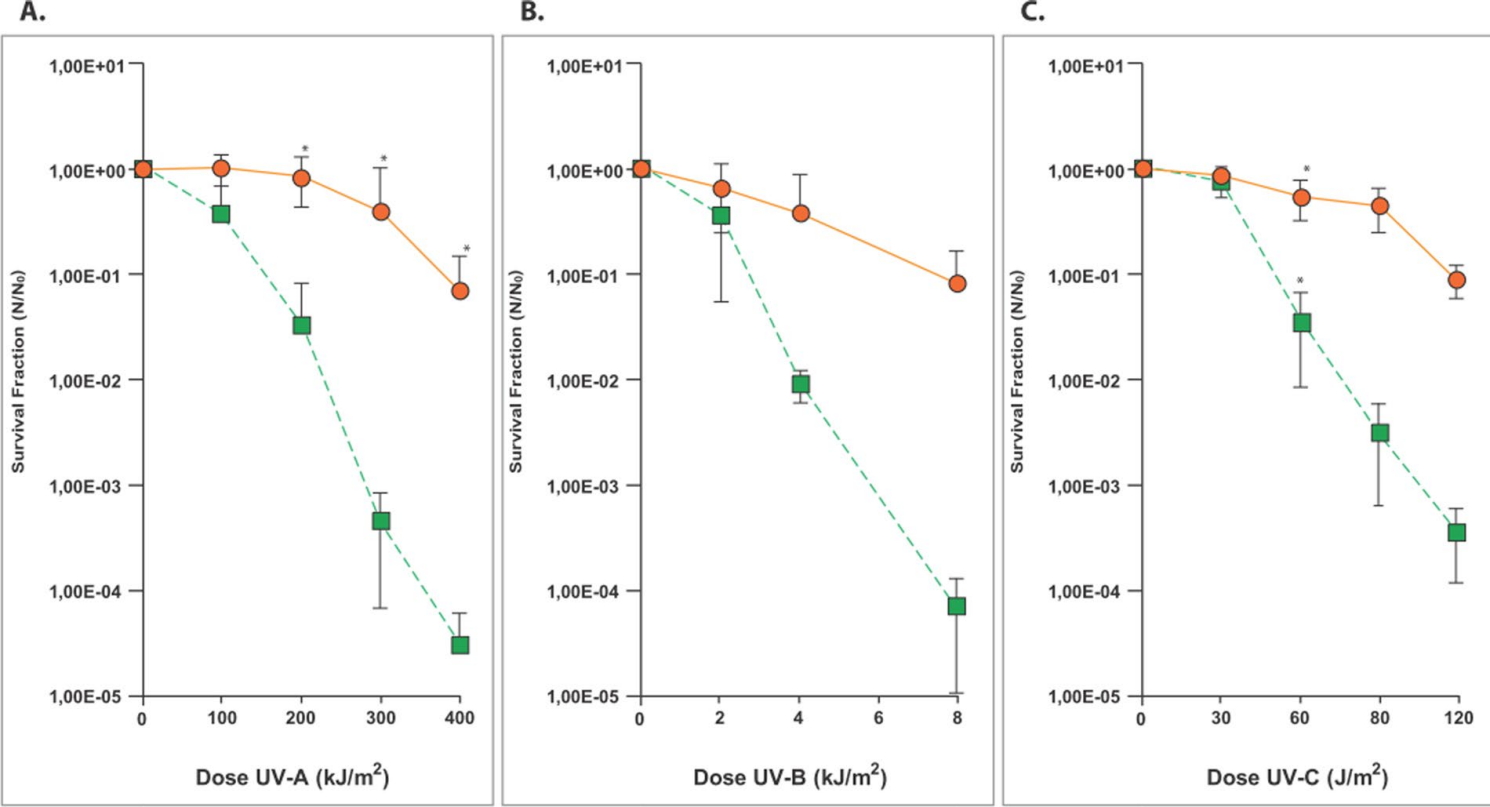
Survival fraction of bacterial species *Microbacterium* sp. *isolate LEMMJ01* () and *E*. *coli* K12A15 strain () after increasing doses of (**A**) UV-A (365 nm), (**B**) UV-B (312 nm) and **C**. UV-C (254 nm) irradiations. For this, stationary growth phase cultures were normalized at the optical densities (O.D.) of 0.1 and 1 for *Microbacterium* sp and *E*. *coli*, respectively. The suspensions of *Microbacterium* sp. *isolate LEMMJ01* and *E*. *coli* K12A15 strain were exposed to UV-A at a dose rate of 40 W/m^2^, UV-B at 15 W/m^2^ and UV-C at 2.5 W/m^2^. Aliquots were withdrawn at different times and plated to determine survival. The asterisks represent a significant difference (*p* < 0.005) between the survival of *Microbacterium* sp. *LEMMJ01* and *E*. *coli* K12A15 after each radiation dose, as assessed by the Student’s *t-*test. Error bars represent the standard deviation of the counts of at least three independent experiments.

In the present work, the *Microbacterium* sp. isolate LEMMJ01 was resistant to high doses of UV radiation when compared with the standard *Escherichia coli* K12A15 strain (Fig. 2). This bacterial model was selected due to the deep knowledge on the induced responses against UV radiation in a way that a better inference on the effects of the pigments could be made^34^. The resistance was determined across the three UV radiation ranges: UV-A (400–315 nm), UV-B (315–280 nm) and UV-C (280-100 nm). Taking into account a normalizing parameter to compare the UV resistance between the two species, the doses leading to lethality of 90% of each bacterial population were compared. As such, the ratios *Microbacterium*/*E*. *coli* was 2.33 for UV-A, 2.67 for UV-B and 2.0 for UV-C. If both are fully proficient in repair mechanisms to remove UV-induced damage from the DNA, then such >2-fold resistance relatively to non-pigmented *E*. *coli* may be ascribed to the anti-oxidant status provided by carotenoids in this *Microbacterium* species. These results pointed out this *Microbacterium* sp. isolate LEMMJ01 to be very resistant to such harmful radiation, most probably due to its adaption to the Antarctic UV regimen. Carotenoids are very efficient physical quenchers of singlet oxygen and scavengers of other reactive oxygen species. Exogenous antioxidants, such as α- and β-carotene, lutein and astaxanthin play important roles in preventing oxidative damages of free radical by this scavenging activity^35^. In addition, these isoprenoids molecules can also act as chemical quenchers undergoing irreversible oxygenation. Even though the molecular mechanisms underlying these reactions are still not fully understood, Lutein, a carotenoid already used in ophthalmology in the treatment of macular degeneration, has been associated with a protective effect of oxidative damage from sunlight, particularly by visible light, by absorbing blue light^36,37^. Lycopene is a carotenoid of greater biological action in the neutralization of singlet oxygen^38^.

These facts demonstrated that the most important bioactivity of carotenoids in living organisms is the antioxidant response and oxidative stress resistance. Their lipophilic nature allows them to penetrate through cellular bilayer membrane. This property is important for the use carotenoids in industry of antioxidants, they can cross the blood-brain barrier, carrying out its biological function also in different regions of human body^35^.

**Figure 3:**
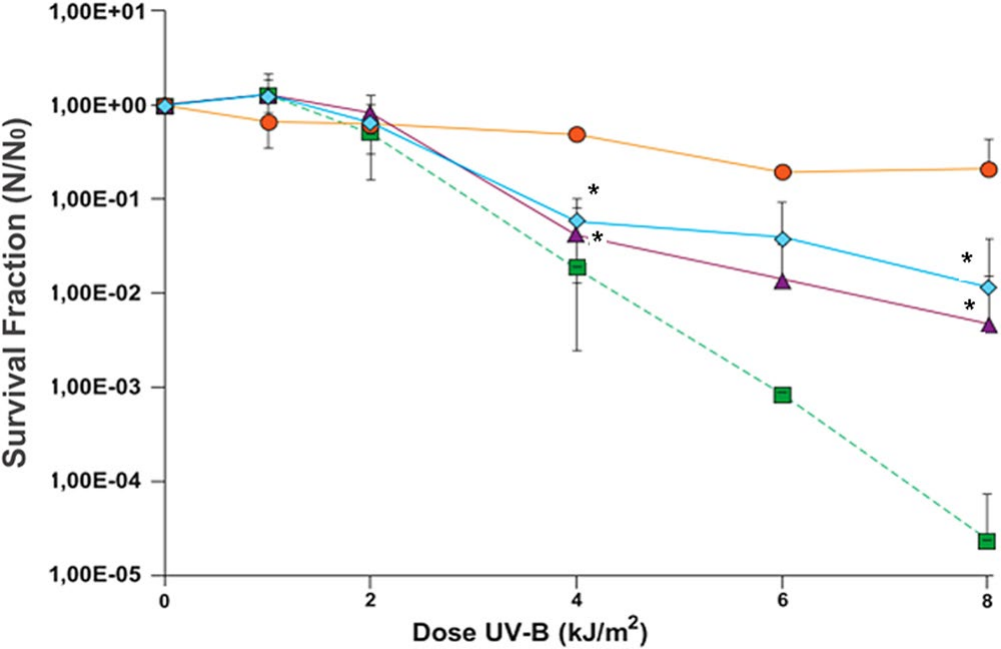
Bacterial survival curve of *Escherichia coli* K12A15 strain () grown overnight in LB medium, with added concentrations of 0.1 mg/ml () and 1 mg/ml () of the pigment produced by *Microbacterium* sp. LEMMJ01 (). To compare the resistance between the two bacterial species with or without the pigment produced by *Microbacterium* sp. against UV-B radiation. Stationary growth phase cultures were irradiated with increasing doses of UV-B (312 nm) and aliquots were withdrawn at different times and plated to determine survival. The asterisks represent a significant difference (P < 0.005) between the survival of *E*. *coli* with and without pigment after the dose of UV-B irradiation as assessed by Student’s *t*-test. Error bars represent the standard deviation of the counts of at least three independent experiments.

On behalf of testing this hypothesis, the crude carotenoid (CC) fraction (0.1 mg/ml and 1 mg/ml) preparations was added to a culture of *E*. *coli* K12A15 strain in LB medium and grown overnight in order to observe the effect on *E*. *coli* survival under UV-B (312 nm) radiation. Aliquots from the cultures were collected at different times and plated on LB medium to determine its survival. The addition of CC fraction at concentrations of either 0.1 or 1 mg/ml to the *E*. *coli* culture led to an expressive protection relatively to the control culture (Fig. 3). Indeed, the carotenoids caused survival against UV-B to raise from ~10^−5^ to 10^−2^, or, regarding the doses required to attain 1% survival, the fraction led UV-B doses to increase 2-fold. Such greater protection induced by the lower CC concentration fraction suggests that there may be not only a structural absorption of the anti-oxidant CC fraction onto the surface of *E*. *coli* K12A15 cells, but also the abovementioned photoabsorpting feature that remained active in an *E*. *coli* strain. Important to mention that similar patterns of UV resistance are found in a well-known extremophile, *Deinococcus radiodurans*, a species in which a family of carotenoids are largely claimed to play a role in its resistance against radiations^39,40^.

The effects of solar radiation, especially UV-B, were evaluated on the growth of Antarctic terrestrial fungi *Geomyces pannorum*, *Phoma herbarum*, *Pythium* sp., *Verticillium* sp., and *Mortierella parvispora*^41^. After 3 h of UV-B continuous radiation exposure, the rates of hyphal extension were reduced in all species. UV-B has been shown to reduce the vegetative growth of two Antarctic plants, the *Colobanthus quitensis* and *Deschampsia antarctica*^42^. Growing on top of the vertical rocks in the Antarctic region, resistant microorganisms can cope with high UV-C doses; the reported lethal doses were 200–300 J/m^2^ and 500–1500 J/m^2^ for *Methylobacterium* isolates and pigmented yeasts respectively^43^. The UV-B resistance of the *Microbacterium* sp. isolate LEMMJ01 is clearly greater than that for the standard *E. coli*, and any dose of radiation in the studied range was able to inflict a significant loss of cell viability.

The discovery of novel anti-UV-B protecting pigments from natural sources is envisaged as of great impact for human health, since it is directly involved with deleterious effects in humans such as sunburn (erythema), tanning and premature aging of the skin, eye damage, and skin cancer induction by causing mutations in DNA as well as suppressing certain antigen-presenting activities of the immune system.

**Figure 4:**
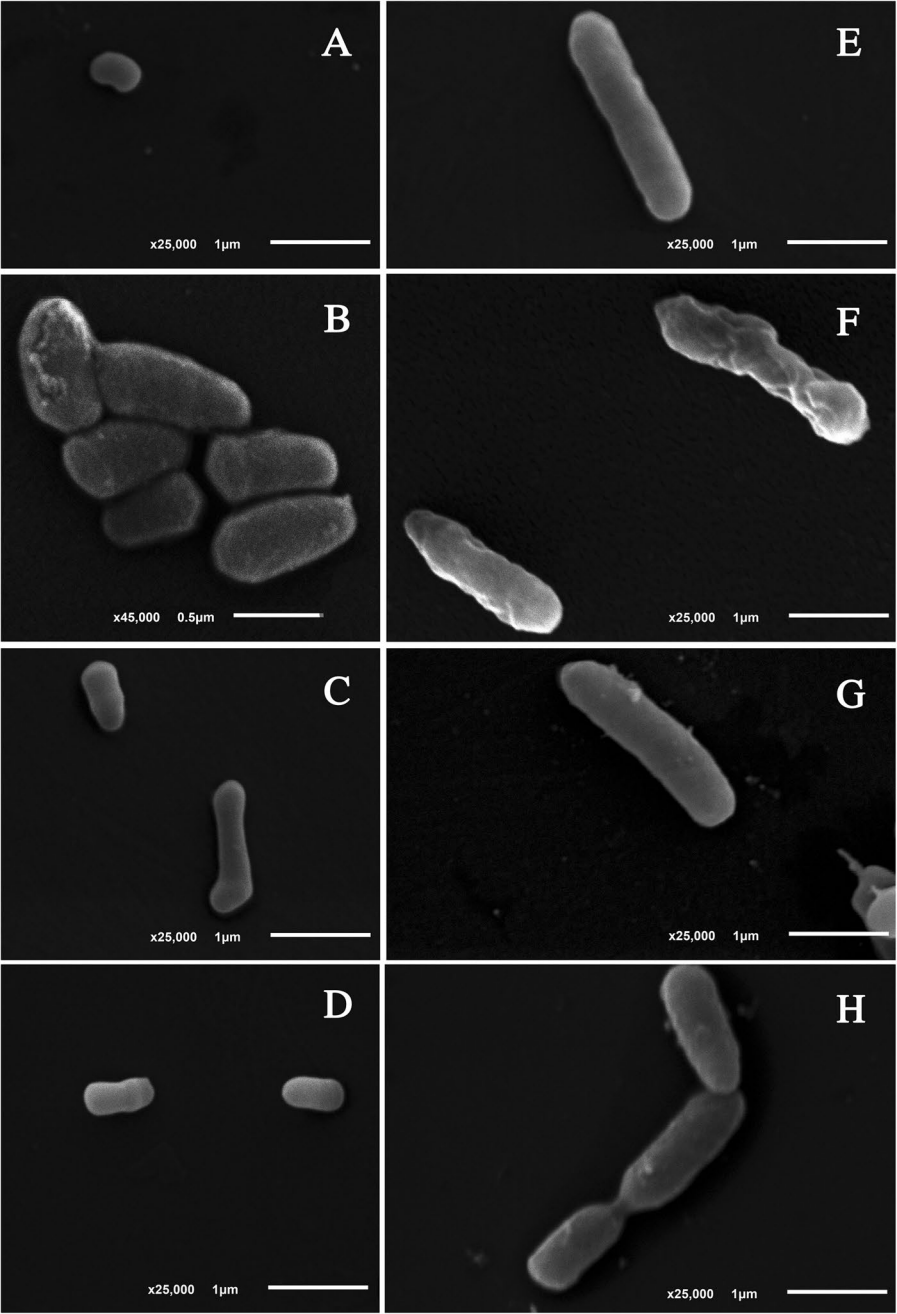
Scanning Electron Microscopy of samples of *Microbacterium* sp. *isolate LEMMJ01* (**A**–**D**) and *E*. *coli* K12A15 strain (**E**–**H**). Non-irradiated samples: A and E (control cells). After UV radiation: (**B**,**F**) (UV-A); (**C**,**G**) (UV-B); (**D**,**H**) (UV-C). The best representative images of main modifications were collected after observation of all fields.

Scanning electron microscopy revealed that non-irradiated cells displayed normal and characteristic shapes for both bacteria: the kidney-like shape for *Microbacterium* sp. (Fig. 4A) and rod-shape cells for *E*. *coli* K12A15 (Fig. 4E). After UV-A radiation, however, severe morphological alterations were observed in *E*. *coli* K12A15, such as a rough surface (Fig. 4F). On their turn, the cells of *Microbacterium* sp. isolate LEMMJ01 maintained the normal cellular structure as observed for the control cells (Fig. 4B). The SEM micrographs taken after UV-B radiation demonstrated no alterations to occur in *Microbacterium* sp. (Fig. 4C). Consistent with its high UV-B sensitivity, membranes of irradiated *E*. *coli* K12A15 samples appeared irregular with superficial leakage of intracellular material (Fig. 4G). No detectable morphological alterations were seen to occur in UV-C irradiated cells of either species (Fig. 4D,H).

### Crude carotenoid fraction characterization

Total carotenoids were measured in the CC fraction that came from *Microbacterium* sp. isolate LEMMJ01, the latter proved to be an excellent source of carotenoids yielding 391.23 mg per liter in 1 gram of fraction of fresh carotenoids, without any or genetic studies to improve this yield (Table 2). This crude carotenoid fraction was analyzed in HPLC-DAD. The main peak, eluting at 3.76, showed a typical carotenoid UV spectrum with λ_max_ at 415, 438 and 468 nm. The peaks eluting at 2.21, and 3.36 min displayed identical absorption bands suggesting structural similarity between the compounds (data not shown). The observed group of UV absorptions can be associated with a polyene chromophore with nine conjugated double bonds as in neurosporene, a carotene found in plants intermediating the biosynthesis of lycopene and other bacterial carotenoids. Trutko *et al*.^25^ detected isoprenoid pigments (carotenoids) in 71 out of 78 strains belonging to 11 genera of the Microbacteriaceae family. According to the authors, eight out of sixteen strains of genus *Microbacterium* were able to synthesize neurosporene^25^.

**Table 2:** Total carotenoids (TC).

Sample (g)	Abs*	TC (mg/l)
*Microbacterium* cells (0,9564)	0,1354	12,9503
CC fraction (0,9551)	4,0866	391,2287

*Abs- absorbance.

**Figure 5:**
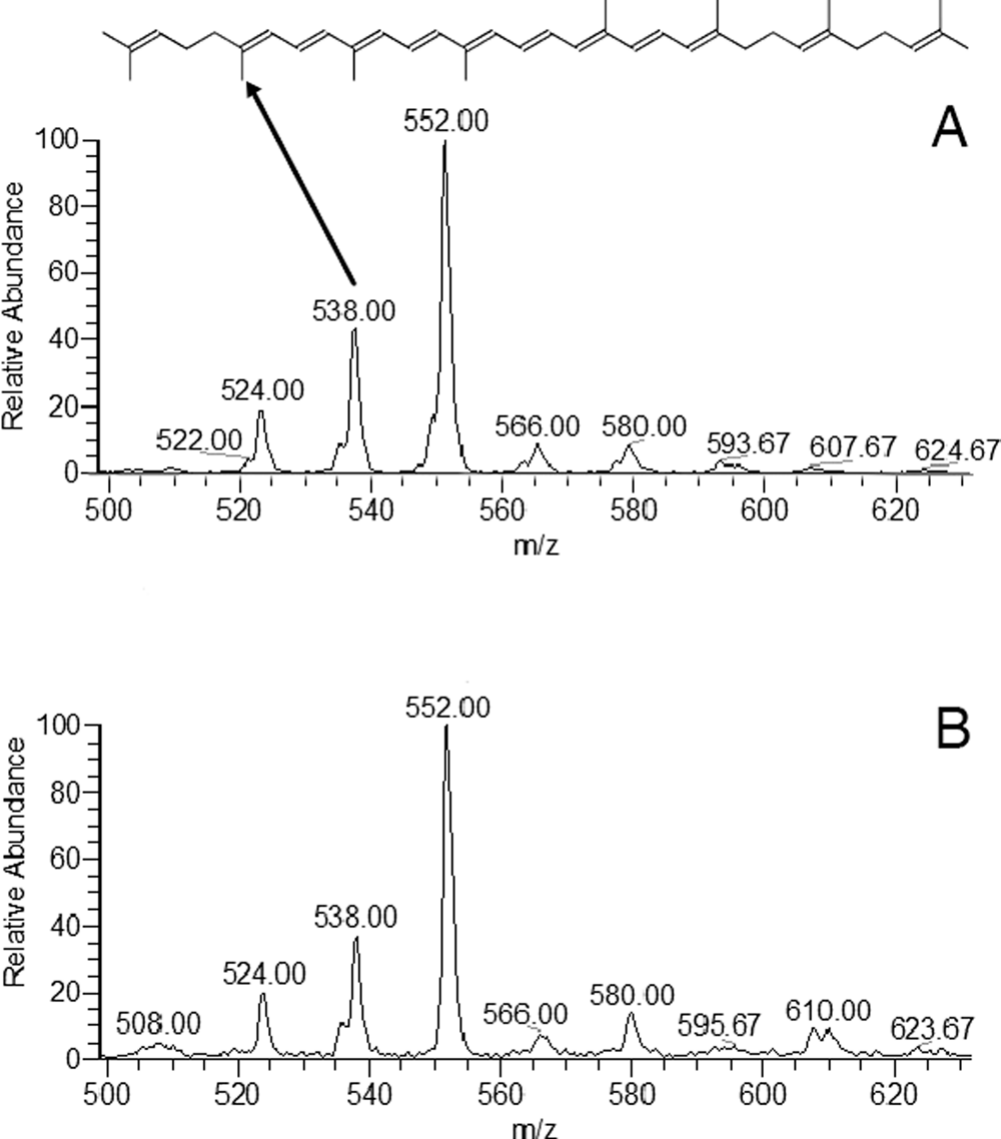
APCI-MS fingerprints obtained in the positive ion mode of: (**A**) CC fraction, (**B**) neurosporene standard (CaroteNature GmbH).

The representative APCI-MS fingerprints of the CC fraction and Neurosporene (CAS No. 502-64-7) standard sample (CaroteNature GmbH) are shown in Fig. 5. The diagnostic ions (m/z 580, 566, 522, 538, and 524) observed in the neurosporene standard are also present in the CC fraction. Neurosporene carotenoid has a molecular formula of C_40_H_58_ with molecular mass of 538.91 g/mol. Antolak *et al*.^44^ and Takaichi *et al*.^45^ when analyzing samples of neurosporene in positive ionization mode also observed the m/z 538 ion^44,45^. In addition to the previously mentioned authors, Ojha *et al*.^28^ also detected neurosporene in the *Microbacterium* genus^28^. However, with the combined information of the UV-Visible spectra mentioned above and the results of APCI-MS, to our knowledge, it was the first time that the extracted sample was compared and matched to a purified neurosporene standard. Thus, our experimental results indicate that the main carotenoids present in the *Microbacterium* sp. are neurosporene and other derived biosynthetic carotenoids as hydroxineurosporene and methoxineurosporene, both with chromophores similar to neurosporene already detected in other actinomycetes^27^. More experiments are on the way to characterize these compounds.

**Figure 6:**
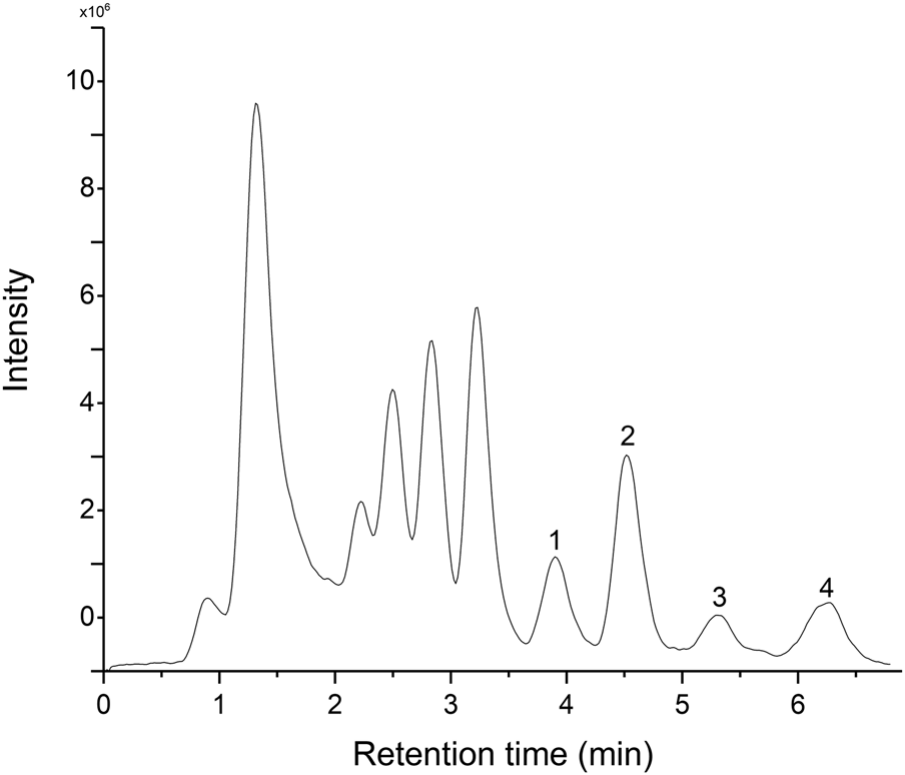
Profile of the HPLC-MS/MS chromatogram of CC fraction. See Table 3 for peak identification.

Alpha-carotene, echinenone, canthaxanthin and astaxanthin for the first time were shown to exist in this species by HPLC-MS/MS (Table 3; Fig. 6). α-carotene, also known as provitamin A, can be distinguished from the isomeric β-carotene, γ-carotene and lycopene by tandem ion mass spectrometry in APCI positive mode. α-carotene differs from β-carotene only by the position of a double bond in one of the terminal rings, an α-ionone moiety. This differentiation is challenging, since most of the ions of β-carotene detected by positive APCI mode are the same as α-carotene (e.g., m/z 137, 413 and 457). However, Van Breemen *et al*.^46^ observed that the m/z 123 fragment corresponds to the α-ionone portion of α-carotene by using APCI in positive mode, thus differentiating it from β-carotene, γ-carotene and lycopene, since this ion in absent under this mode. This evidence is in agreement with the results observed in this work (Table 3), where the precursor ions m/z 537.486 (peak no. 1- Table 3, Fig. 6) were fragmented, generating the ions m/z 123, 137, 177 and 413, which according to Van Breemen *et al*.^46^ correspond to α-carotene carotenoid. Based on peak no. 2 (Table 3, Fig. 6) the presence of the compound echinenone is proposed. This work fragmented the precursor ion m/z 551.4252 which gave the fragments m/z 133, 203, 255, 495 and 536 corresponding to 71% of the ions obtained by fragmentation of echinenone in positive APCI by Rivera *et al*.^47^. The presence of canthaxanthin was confirmed at peak no. 3 (Table 3, Fig. 6). According to Qiu *et al*.^48^ the carotenoid all-trans-canthaxanthin when fragmented in m/z 565.7 generates the ions m/z 547.4 [M + H-18]+, 363.6 and 203.5, which correspond to 100% of the observed fragments in this work upon fragmentation of the precursor ion m/z 565.5186 [M + H]+. An ion with m/z 579 (peak no. 4- Table 3, Fig. 6) was detected and was fragmented generating the ions m/z 285, 379 and 561, it was assigned as the compound astaxanthin, based on the work of Rivera *et al*.^47^ that analyzed astaxanthin in APCI positive mode by the fragmentation of precursor ion m/z 597, and observed the formation of fragments m/z 579 [M + H-18], 561 [M-18-18], 505 [M-92], 379 and 285.

**Table 3:** Chromatographic and mass characteristics of carotenoids obtained from crude carotenoid fraction (CC fraction) by HPLC-MS/MS.

Peak no.	Rt (min)	Measured (m/z)	Molecular formula	Theorical (m/z)	Fragment ions (m/z)	Proposed compound
1	3.93	537.4860	C_40_H_56_	537.4460	123,137,177,413	alpha-carotene
2	4.53	551.5047	C_40_H_54_O	551.4252	133,203,255,495,536	echinenone
3	5.32	565.5186	C_40_H_52_O_2_	565.4045	547,363,203	canthaxanthin
4	6.26	579.5359	C_40_H_52_O_4_	596.3865	285,379,561	astaxanthin[M + H- 18]+

Peak no. = peak number; Rt = retention time.

Neurosporene is a tetraterpene molecule that belongs to the widely commercialized carotenoid family. Unlike other carotenoids microbial production of neurosporene has been rarely described, probably because neurosporene is often a bound intermediate of lycopene synthase. An engineered *E*. *coli* cells expressing the lycopene biosynthesis pathway with a modification in the isopentenyl phosphate kinases- IPK, improved the neurosporene content by more than 45-fold. IPK is a enzyme of the mevalonate pathway which is predominantly found in eukaryotes and archaea^49^. The fact that neurosporene is the most abundant carotenoid in *Microbacterium* sp. studied in this work is a differentiate characteristic when compared to other microorganisms. The red pigment astaxanthin possesses strong scavenging activity against free radicals and other prooxidant molecules, protecting lipid bilayer from peroxidation. This capacity 10-fold greater than other carotenoids is due to polar ionic rings and non-polar conjugated carbon–carbon bonds. Astaxanthin is also well-known to prevent peroxidation at the lipid membrane level with efficiency. During quenching activity astaxanthin molecule is not subjected to chemical modification and can be active for many cycles in addition astaxanthin was able to reduce proinflammatory cytokines, such as IL-1β, IL-6 and TNF-α, protect against H_2_O_2_^35^.

In Antarctica microorganisms are exposed to several conditions that trigger the generation of reactive oxygen species, such as high UV radiation. Scavenging of reactive oxygen species is considered a key mechanism of action underlying the photobiological protective activity of carotenoids. The presence of a mixture of carotenoinds such as neurosporene, alpha-carotene, echinenone, canthaxanthin and astaxanthin in Microbacterium could represent an antioxidant defence system working with synergistic effect against the high solar incidence in Antartic continent^35^. This diversity of carotenoids species found in *Microbacterium* sp. could be related with the high summertime UV exposure in the soil Antarctic region from which the species was isolated. Carotenoids absorb UV light and prevent direct damage of a number of cellular targets. In this context, a combination of antioxidant compounds (with β-carotene, as the main component, α-tocopherol and ascorbic acid) have a strong antioxidant effects in cultured human fibroblasts, irradiated with UV-A light (20 J/cm)^50^. Due to their strong anti-oxidant properties, carotenoids such as β-carotene and canthaxanthin have been employed as dietary supplementation in order to protect human skin against UV radiation^51,52^.

### Phototoxicity and cytotoxicity analysis of CC fraction

**Figure 7:**
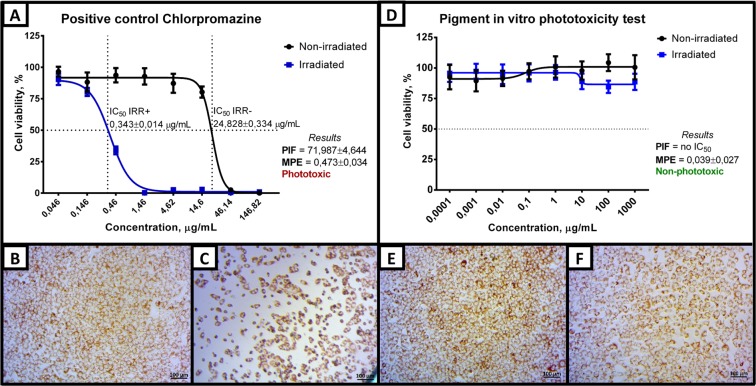
Assessments of phototoxicity of CC fraction. (**A**). Dose-response curve and results for chlorpromazine. After simulated sunlight, the lethality of Balb 3T3 cells A31 increases. Scores of PIF and MPE classify chlorpromazine as phototoxic and the OECD TG 432 defines the following reference parameters: IC_50_ (Irr+) = 0.1 to 2.0 µg/ml, IC_50_ (Irr−) = 7.0 to 90.0 µg/ml and PIF > 6. (**B**,**C**) Representative optical micrograph of the neutral red uptake in samples treated with 0.46 µg/ml chlorpromazine (non-irradiated and irradiated, respectively). (**D**) Dose-response curve and results for the CC fraction induced-phototoxicity. The presence of simulated sunlight was not able to reduce significantly the cell viability to the maximum concentration of 1 mg/ml allowed by the test. According to the MPE score, the CC fraction can be classified as non-phototoxic (MPE < 0.1). (**E**,**F**) Representative optical micrograph of the neutral red uptake after treatment with 1.0 mg/ml CC fraction (not irradiated and irradiated, respectively). Error bar represents a confidence interval of 95% (n = 12, of two independent experiments of n = 6) of a t-student distribution. Cells were irradiated for 50 min in irradiance of 1.7 mW/cm². The viability of the irradiated control is more than 80% relatively to the non-irradiated control, thus exposure to simulated sunlight alone does not cause significant baseline cytotoxicity in the test system.

Determination of phototoxicity in cell culture models is a valuable alternative method as a first screening for test compounds before they follow to tests with animals. The test evaluates the light-induced response when a photoreactive substance is activated by solar light producing cytotoxic chemicals potentially harmful to human skin, with consequent skin irritation. UV-B and UV-A are responsible for the manifestation of phototoxicity. Phototoxicity was performed in Balb 3T3 clone A31 mouse cells, with the positive control consisting in the exposure to chlorpromazine. Under Simulated Solar Light, after irradiation for 50 min under a dose rate of 1.7 mW/cm² (total dose = 5 J/cm²), the PIF and MPE indexes increased to 71.987 and 0.473, respectively, in chlorpromazine-treated Balb 3T3 clone A31 cells. This classifies chlorpromazine as phototoxic according the OECD Test Guideline N° 432^53,54^. In contrast, the IC_50_ cannot be obtained for the CC fraction, but the MPE of 0.039 indicated that the CC fractions from *Microbacterium* sp. LEMMJ01 were not phototoxic at all (Fig. 7).

**Figure 8:**
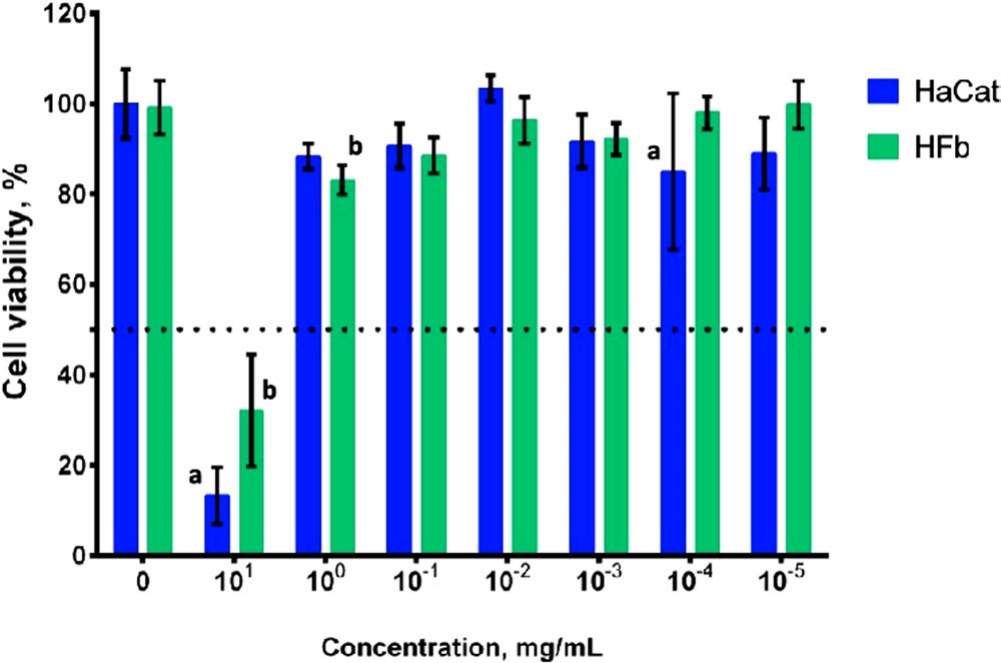
CC fraction cytotoxicity in human keratinocytes (HaCat) and fibroblasts (HFb). Error bars represent a confidence interval of 95% (n = 12, two independent experiments of n = 6) of a *t*-student distribution. (*) significant statistic difference (p < 0.05) between cell lines. (a) significant statistic difference (p < 0.05) in relation to the control group (no treatment) of HaCaT. (b) Significant statistic difference (p < 0.05) in relation to the control group (no treatment) of HFF.

The cytotoxicity of the CC fraction was analyzed in keratinocytes HaCaT (BCRJ N° 0341) and fibroblast HFF-1 (ATCC SCRC-10410) cell line. The concentration leading to 50% cytotoxicity (CC_50_) value for the two cell lines was greater than 10.000 μg/ml, the highest concentration used in the experiment (Fig. 8). When analyzing the data normalized by 100% of metabolism of control cells, it was possible to observe that no concentration equals or is lower than 1000 μg/ml, whose were not able to reduce at least 50% of the metabolic activity for both cell lines. This indicates that, regarding the MTT method, there was no cytotoxic effect of CC fraction at the concentrations used for both cell lines. Additionally, the viability of the two cell lines was not altered by the addition of the CC fraction.

Solar UV radiation favors the generation of free radicals leading to a greater or lesser extent, photoaging, photoimunio suppression and photocarcinogenesis. (UV) radiation and UV-B range (290–320 nm) acts mainly on keratinocytes and for UV-A band (320–400 nm the major targets are the melanocytes and fibroblasts, generating reactive oxygen species (ROS) among other effects. The main ROS are the hydroxyl radicals (HO^•^) and superoxide (O_2_^•−^), peroxyl and alkoxyl radicals (RO_2_^•^ and RO^•^) the singlet oxygen (^1^O_2_), as well as hydrogen peroxide (H_2_O_2_) and organic peroxides (ROOH^•^). In addition to direct damage to molecules such as lipids, aminoacids and DNA, ROS can activate enzymatic and non-enzymatic cellular responses^37^. Skin is exposed to chemical and physical factors such as UV radiation and represents a fundamental barrier between our body and the external environment. Besides ROS formation, epidermal and dermal damages, photosensitivity disorders are related to UV radiation. It has been demonstrated that lycopene, lutein, α-, γ-, β-carotene, zeaxanthin and their isomers are carotenoids present in human skin are part of the antioxidant defense system^55^. Carotenoids are a class of antioxidants able to delay, reduce, modify, prevent or remove oxidative damage in different ways and at different levels. In this context microbial carotenoids is a source of these molecules with potential use in skin photoprotection. Our results, analyses on the phototoxic and cytotoxic effects of the carotenoids present in the studied CC fraction from *Microbacterium* sp. address to its safe use as additives in sunscreens formulations.

Concluding, in this study, the actinomycete *Microbacterium* sp. LEMMJ01 isolated from ornithogenic Antarctic soils harbors high resistance against UV- radiations, mainly UV-B. The analyses on carotenoids composition in the crude carotenoid fraction (CC fraction) revealed the existence of neurosporene, α-carotene, echinenone, canthaxanthin and astaxanthin with a significant photoprotective effect. No phototoxic or cytotoxic effects were seen to be induced by the CC fraction in mouse cells or in human keratinocytes and fibroblasts. These results point out the *Microbacterium* sp. isolate LEMMJ01 as a valuable source of carotenoids (391.23 mg per liter) and that its CC fraction has a potential use for application in skin photoprotection.

## Methods

### Area of soil sampling

The *Microbacterium* sp. isolate LEMMJ01 and other bacteria were isolated from soil samples collected close to the Arctowsky Polish Station near an Adelie Penguin (*Pygoscelis adeliae*) nest (62°09′790″ S;58°27′687″W), in King George Island, belonging to the South Shetland Archipelago, located Northwest of the Antarctic Peninsula during the austral summer of 2009/2010. All procedures were carried out with the support of the Brazilian Antarctic Program (PROANTAR). Temperatures range between −3 °C to 8 °C in the southern summer, with glaciers covering 90% of the island, while the ice-free locations are in the coastal zone where soils undergo freezing and thawing cycles. The Antarctic soils characterized as ornithogenic are located in areas with penguin colonies, and thus are rich in organic matter. Approximately 500 g of soil (0–10 cm deep) were collected, transferred to sterile plastic bags (Whirl Pack®) and then immediately conditioned in the laboratory of the Brazilian Antarctic Station Commander Ferraz (EACF) in a refrigerator at 4 °C until being transported to Brazil.

### Isolation and cultivation of bacterial species

The soil sample was transferred to an Erlenmeyer flask where it was suspended in 45 ml of saline solution (0.85%), 5 g of glass beads, and was homogenized on an orbital shaker for 1 h. After this, serial dilutions (1:10), from 10^−1^ to 10^−3^ were seeded by the spread plate technique, in duplicate, onto Petri dishes containing solid LB medium^56^. The plates were incubated for 20 days in an incubator at 4 °C, 10 days at 12 °C followed by 48 h at 28 °C until colonies appeared which were counted later. Plates with 10 to 50 colonies (diluted by 10^−2^), were characterized in terms of colony morphotypes, and isolated on Petri dishes containing solid LB medium by the depletion technique, to purify the isolates. The purity of cultures was observed by homogeneity of each morphotype and by the Gram staining technique. Subsequently, isolates were picked up and cultured under the same conditions until optical density (OD) 600 nm of 0.5 was reached. Pigmented colonies cultured at 28 °C were preselected for this study.

### Molecular identification of bacterial isolates and phylogenetic analysis

Identification of the selected isolate was performed by extracting genomic DNA with the commercial Wizard^®^ Genomic DNA Purification Kit (PROMEGA) and quantified using QUBIT fluorometer (Thermo Fisher Scientific). DNA purity and quality were evaluated by 1% agarose gel electrophoresis containing SYBR^®^ Safe DNA gel stain (Life Technologies™) at 90 V in 0.5X TBE buffer for 1 h, being visualized by translumination under ultraviolet light. The obtained DNA was subjected to amplification of the 16S RNA ribosomal subunit (*rrs* gene) by PCR using the universal primers 27 f (5′-AGA GTT TGA TCM TGG CTC AG-3′) and 1492r (5′-TAC GGY TAC CTT GTT ACG ACT T-3′). Amplicons generated by PCR were purified using a PCR Purification Kit (Qiagen) for subsequent automated sequencing using a MEGABACE DNA Analysis System 500 (GE Healthcare). The sequencing was performed using the primers 27 F (5′-AGA GTT TGA TCA TGG CTC AG-3′), 1492 R (5′-GTT TAC CTT GTT ACG ACT T-3′), 532 F (5′-CGT GCC AGC AGC CGC GGT AA-3′) and 907 R (5′-CCG TCA ATT CMT TTG AGT TT-3′)^57^.

The quality of the obtained sequences was assessed through the Phred program^58^ and the high-quality sequences of each isolate were assembled with the MEGA 7.0 program. The pooled sequences were queried against all known DNA sequences deposited in non-redundant databases from the Ribosomal Database Project (RDP). Alignment of all sequences and construction of the tree were done with the help of the ClustalW tool, available in the MEGA 7.0 program, adopting the Neighbor-Joining algorithm and the bootstrap value of 1000 replicates. To build the root of the phylogenetic tree, the *Desulfurococcus fermentans* archaea was selected as the external group.

Molecular phylogenetic analysis was done by Maximum Likelihood method comparing *Microbacterium* sp. isolate LEMMJ01 with the nearest hit in RDP. The evolutionary history was inferred by using the Maximum Likelihood method based on the Tamura-Nei model^59^. Evolutionary analyses were conducted in MEGA7^60^.

### Sample preparation and response to UV radiation

The cultures of the selected bacteria, *Microbacterium* sp. isolate LEMMJ01 and the reference strain *E*. *coli* wild type K12A15, were cultivated and maintained in LB medium. The cells were prepared for irradiation by growing an inoculum overnight at 28 °C under shaking at 160 rpm (Environmental Incubator Shaker - G24) until they reached the stationary phase (10^9^ cells/ml). Cultures were harvested at 6,000 × *g* at 4 °C and the cell pellets were washed twice with M9 buffer and resuspended in the same buffer^61^. Cultures of *Microbacterium* sp. and *E*. *coli*, were subsequently adjusted to reach the optical densities (O.D. = 600 nm), and then exposed to UV radiation. Cell suspensions were irradiated with UV-A emitted by a VL212 lamp (emission peak at 365 nm; Vilber Lourmat); a VL215 lamp (peak at 312 nm; Vilber Lourmat) was the UV-B source; and the UV-C source was a General Electric 15-W germicidal lamp G15T8 (254 nm). To determine the total doses of UV-A, UV-B and UV-C a Model VLX-3-W dosimeter (Vilber Lourmat, France) was used, with appropriate photocells. After each dose, aliquots were serially diluted in M9 buffer and plated on solid LB nutrient 1.5% agar (Difco Laboratories) plates. Cell surviving fractions were determined by the ratio between the number of remaining cells after each UV dose and the initial number of viable cells. Briefly, after 24–48 h incubation at 28 °C the colony-forming units were counted and multiplied by the corresponding dilution factor in order to estimate the average number of survivors (N). This number was divided by the value corresponding to the non-irradiated control (N_0_), which yielded a survival rate (N/N_0_) for each dose. All experiments were conducted in triplicates and plots represent the mean ± standard error of these replicates.

### SEM preparation samples and observation

Approximately 0.5 h after irradiation, the aqueous solutions of *E*. *coli* K12A15 and *Microbacterium* sp. isolate LEMMJ01 cultures were put into sterile Petri dishes. A fixative solution was added to the cell suspension, containing 2% osmium tetroxide, 12% glutaraldehyde and 0.2 m cacodylate buffer following the proportions 1 ml; 2 ml; 1 ml for 30 minutes, according to Silva-Neto *et al*.^62^. Circular glass coverslips previously sterilized under UV light for 30 minutes were placed inside the dishes to allow bacterial cells to attach to their surfaces. The coverslips were then removed from the solution and put into holders and then dehydrated in an alcoholic series. They were transferred to containers with ethylic alcohol in successive and increasing concentrations of 30%, 50%, 70%, 85%, 90%, for 10 minutes each and ultimately alcohol 100% for 20 minutes. At the end of the dehydration in alcoholic series, the coverslips were transferred to a Balzers CPD 30 critical dryer, to complete the drying process by replacing the absolute ethylic alcohol for liquid CO_2_, under low temperature (around 8 °C) and its posterior evaporation between 20 °C and 38 °C. After the dehydration stage, the coverslips were fixed onto metallic stubs using a double-sided tape. They were then transferred to a Leica EM SCD050 metallizer to be gold-coated, by applying a 40-mA electric current for 90 seconds, sputtering a 25 nm thick gold film. A scanning electronic microscope, JEOL JSM-6510 was used to observe the bacteria and obtain the electron micrographs.

### Extraction of the Crude Carotenoid (CC) fraction

Organic extracts were prepared with methanol. Briefly, dried *Microbacterium* sp. isolate LEMMJ01 cells (2 g) were extracted with methanol (16 ml). The suspension was centrifuged at 10.000 × g for 10 min at 4 °C. The extraction procedure was repeated twice. After centrifugation, the supernatants were combined and evaporated to obtain the CC fraction, At the end, it was stored in a freezer at −80 °C. Before the analysis, the CC fraction was totally solubilized in methanol 80%”.

### Measurement of the concentration of total carotenoids

This assay was performed by directly measuring the absorbance of the methanolic fraction, the CC fraction using UV–Visible spectrophotometer at 450 nm. Results were calculated using equation expressed below where A_total_ = absorbance_,_ m = mass, v = extract volume; A^1%^ = coefficient specific of absorption of mixtures (2500)^63^.

$$\frac{{{\rm{A}}}_{{\rm{total}}}\times {{\rm{v}}}_{({\rm{mL}})}\times {10}^{{4}^{1{\rm{cm}}}}}{{{\rm{A}}}^{1 \% }\times {\rm{m}}({\rm{g}})}$$

### High performance liquid chromatography with diode array detection (HPLC-DAD) analysis

The crude carotenoid fraction was subjected to HPLC in the reversed phase mode using a Waters YMC C30 column (250 × 4.6 mm, 5 μm internal diameter), at room temperature, with a flow rate of 1 ml/min, and injections of 20 μl. The HPLC mobile phase consisted of a linear gradient of methyl tert-butyl ether in methanol. The diode array detector was set at 450 nm.

### Atmospheric Pressure Chemical Ionization Mass spectrometry (APCI- MS) analysis

All experiments were performed with HPLC grade methanol and water was purified by a Milli-Q gradient system (Millipore, Milford, MA, USA). Stock solutions of Neurosporene CAS No. 502-64-7 (CaroteNature GmbH, Ostermundigen, Switzerland) standard compound (1 mg/ml) and solutions (1 mg/ml) of the CC fraction were prepared and stored in methanol at 4 °C. All mass spectra of infused sample solutions were acquired in a continuous monitoring mode (Thermo LCQ Fleet Tune application) using an LCQ Fleet ion-trap mass spectrometer (Thermo LCQ Fleet, San Jose, CA, USA) with an Atmospheric Pressure Chemical Ionization (APCI) interface and running in the positive ion mode to perform APCI-MS analyses. The data were acquired in scan mode using an m/z range of 100–1000. The APCI-MS parameters were: positive polarity; Vaporizer Temp (°C): 450; Sheath Gas Flow Rate (arb): 10; Aux Gas Flow Rate (arb): 5; Sweep Gas Flow Rate (arb): 0; Discharge Current (µA): 4.50; Capillary Temp (°C): 400; Capillary Voltage (V): 13; Tube Lens (V): 75. Nitrogen (>99% purity) was used as nebulizing gas. Data acquisition was carried out with Thermo Xcalibur 2.2 software.

### High performance liquid chromatography coupled to mass spectrometry (HPLC-MS/MS) analysis

The analysis of the crude carotenoid fraction with high performance liquid chromatography coupled to mass spectrometry (HPLC-MS/MS) was performed in a MicrOTOF Bruker^®^ Daltonics GmbH mass spectrometer using APCI ionization coupled to an Agilent® Infinity 1260 chromatograph.

Chromatography: ACE-3 C18 column (pre-column), dimensions 100 × 2.1 mm, 2.7 μm, at 25 °C, flow rate of 0.4 ml/min at wavelengths 408, 440 and 450 nm, using a linear gradient of methanol in MTBE as the mobile phase (step A: methanol, step B: MTBE, gradient: 0 min = 0% B, 20 min = 20% B, 24 min = 50% B, 30 min. = 100% B). Mass spectrometry conditions: APCI ionization, positive mode, flow 0.240 ml/h, scanning 120–1200 m/z, capillary 3500 V, nebulizer 0.6 Bar, N_2_ temperature: 180 °C and flow rate of N_2_: 5 l/min. The identification of the substances was carried out based on the fragmentation profiles in comparison with literature data. The mass measurement was made at a resolution of 20.000.

### Phototoxicity assay

The CC fraction containing the pigment was used in this phototoxicity test. The Balb 3T3 clone A31 cells from the American Type Culture Collection (ATCC) was cultivated and expanded in DMEM high glucose medium supplemented with 10% fetal bovine serum (complete medium), until 80% of confluence. The phototoxicity of CC fraction was evaluated according to the OECD Test Guideline N° 432^53^, using 7.5 – 2.4 × 10^−2^ µg/ml of chlorpromazine as the positive control. Briefly, cells were seeded in two 96-well plates for 24 h ± 2 h incubation and then treated with 8 decimal geometric concentration series of the CC fraction diluted in Hank’s Balanced Salt Solution (HBSS). The plates were incubated for 1 h in the dark, and subsequently, one plate was irradiated for 50 min under a dose rate of 1.7 mW/cm² (total dose = 5J/cm²) and the other plate was kept in the dark for the same time. The irradiation was done using a Q-Sun XE-1-BC Xenon Test Chamber equipped with xenon arc lamp and Daylight optical filter (Simulated Solar Light). Afterwards, the cells were incubated in complete medium for 20 h ± 2 h. The relative cellular viability was estimated by Neutral Red Uptake: the cells was wash with DPBS-A, incubate for 3 h in neutral red solution, then extracted with a solution of 50% ethanol +49% distillated water +1% acetic acid and absorbance was determined at 540 nm. The photoirritation factor (PIF) or the mean photo effect (MPE) scores classify a chemical as phototoxic (PIF > 5 or MPE > 0.15), as probably phototoxic (2 < PIF < 5 or 0.10 < MPE < 0.15) or as none phototoxic (PIF < 2 or MPE < 0.10). The first score is used to classify chemicals with IC_50_ under both conditions, with (+Irr) and without (−Irr) irradiation and the second one is used to classify chemicals that do not meet these criteria.

### CC fraction cytotoxicity in human keratinocytes and fibroblasts

The human keratinocyte cell line (HaCaT; BCRJ N° 0341), from the Rio de Janeiro Cell Bank (BCRJ), was cultivated and expanded in Dulbecco’s modified Eagle’s medium (DMEM; BioWhittaker, Lonza, USA) with 10% fetal bovine serum (FBS; Gibco, Life technologies, USA), 4.5 mg/ml glucose, 4 mM l-glutamine, 1 mM sodium pyruvate, 100 UI/ml penicillin and 100 µg/ml streptomycin (Sigma-Aldrich, USA). The human fibroblast cell line (HFF-1; ATCC SCRC-1041), from the American Type Culture Collection (ATCC), was cultivated and expanded in DMEM with 10% FBS, 1.0 mg/ml glucose, 4 mM l-glutamine, 1 mM sodium pyruvate, 100 UI/ml penicillin and 100 µg/ml streptomycin. Cells were incubated at 37 °C ± 1 °C in a humidified atmosphere (90% ± 10%) containing 5% ± 0.5% CO_2_. HaCaT and HFF-1 cells were seeded in 100 µl aliquots per well at a density of 1.0 × 10^4^ in two 96-well plates and incubated for 24 h ± 2 h. A logarithmic series (1:10) of seven dilutions were prepared in complete medium from the stock solution of 10 mg/ml CC fraction in complete medium, *i*.*e*., 10^1^–10^−5^ mg/ml range. The medium in 96-well plates was decanted and the cells were treated (n = 6) with 100 µl of eight concentrations of the CC fraction for 48 h ± 2 h. The relative cellular viability was estimated by MTT reduction. The medium was removed from the plates, then 100 µl of 0.25 mg/ml MTT in complete medium was added in each well and incubated for 3 h ± 10 min. Afterwards, the MTT solution was decanted and blue formazan crystals were extracted with 100 µl of DMSO. The absorbance was read at a wavelength of 570 nm after 20 min of extraction in an orbital shaker protected from light.

### Statistical analysis

Databases and statistical analyses were performed by means of the Microsoft Excel 2010 and GrafhPad Prism^®^5 softwares in the case of bacterial survival against UV radiation. Statistical tests to compare data on resistance to UV radiation were applied after they were analyzed in relation to the Gaussian distribution by the Kolmogorov-Smirnov test. Afterwards, the Student’s *t*-Test was applied to the unpaired samples, when the estimated parameter presented normal distribution in the two groups under comparison. When at least one of the groups did not followed normal distribution, the Mann-Whitney test was applied. Results with p ≤ 0.05 were considered statistically significant.

The efficacy of nanoemulsions were expressed as the mean ± SD or standard error by means of the software Origin Pro 8 (OriginLab, USA) and considered statistically significant if p < 0.05.

For data analysis of phototoxicity tests, data were arranged in Microsoft Office^®^ Excel^™^ 2016 spreadsheets. To calculate PIF and MPE, the Phototox 2.0 program was made available free of charge by the OECD at: http://www.oecd.org/. Dose-response plots were generated by the GraphPad Prism^®^6 program using the proprietary algorithm for 4-parameter nonlinear regression (4-parameter Hill Logistic Function). For the evaluation of statistical difference, considering α = 0.05, the one-way ANOVA test was used for the values of PIF, MPE, IC50 IRR+, and IC50 IRR−.

## Data Availability

Data used in this manuscript will be available to the public.
